# An HIV-1 Mini Vaccine Induced Long-lived Cellular and Humoral Immune Responses

**Published:** 2015

**Authors:** Mehdi Mahdavi, Massoumeh Ebtekar, Zuhair Mohammad Hassan, Sobhan Faezi, Hamidreza Khorram Khorshid, Morteza Taghizadeh, Keyhan Azadmanesh

**Affiliations:** 1 *Department of Immunology, Pasteur Institute of Iran, Tehran, Iran.*; 2 *Department of Immunology, Faculty of Medical Sciences, Tarbiat Modares University, Tehran, Iran.*; 3 *Department of Mycobacteriology and Pulmonary Research, Pasteur Institute of Iran, Tehran, Iran.*; 4 *Genetics Research Centre, University of Social Welfare and Rehabilitation Sciences, Tehran, Iran.*; 5 *Department of Medical Virology, Razi Vaccine and Serum Research Institute, Karaj, Iran.*; 6 *Department of Virology, Pasteur Institute of Iran, Tehran, Iran.*

**Keywords:** BALB/c mice, HIV-1, long-lived immune responses, P24-Nef fusion peptide

## Abstract

Memory formation is the most important aspect of a vaccine which can guarantee long-lasting immunity and protection. The main aim of the present study was to evaluate the memory immune responses after immunization with a mini vaccine. Mice were immunized with human immunodeficiency virus-1 P24-Nef fusion peptide and then cellular and humoral immune responses were evaluated. In order to determine long-lived memory, immune responses were monitored for 20 weeks after final immunization. The results showed that the candidate vaccine induced proliferation and cytotoxic T lymphocyte responses and shifted cytokine patterns to T helper-1 profile. Evaluation of humoral immune responses also showed an increase in total peptide specific-IgG titer and a shift to IgG2a humoral response. Monitoring of immune responses at weeks 4, 12 and 20 after last immunization showed that immunologic parameters have been sustained for 20 weeks. Our findings support the notion that long-lived memory responses were achieved using a mini vaccine immunization.

Making an efficient human immunode-ficiency virus-1 (HIV-1) vaccine is still a puzzle in today's world. Even though many candidate vaccines were developed to fight against this infection, but none of them have yet displayed any protective effect in human clinical trials (1-3). HIV-1 vaccines in experimental trial are targeting broad CD8+ cytotoxic T lymphocyte responses with the hope that they can prevent or reduce initial viral burden after infection and dissemination of virus from mucosal sites into the systemic circulation (4). Current studies have concentrated on multi-epitopic vaccines with highly immuno-genic properties (5). Utilizing immuno-genic and conserved epitopes from the critical proteins of HIV-1 is a viable approach to attain an immunogenic and effective immune response (5, 6). Herein, clustering of epitopes from different parts of structural and regulatory proteins of HIV particle could be of use (7, 8). The construction of immunodominant epitopes from HIV-1 is a strategy that could enable a strong stimulation of humoral and cellular immune responses. As a result, now it is accepted that targeting of both humoral and cellular immune responses are needed for induction of protection against HIV infection (9). The critical role of CD8+ T cells in control of viral replication in human and non human primate models is unavoidable (10). In fact, the control of viral load initially needs the induction of strong cytotoxic T lymphocyte responses (10, 11). Targeting of the cytotoxic T lymphocyte (CTL) response to certain epitopes of HIV proteins has been shown to be useful in controlling infection (12). Studies in HIV infected patients show that CTL responses directed against HIV P24 and Nef proteins, correlate with disease progression and the intensity of the immune response is directly linked to the prevention of illness progression (13). Nef is a regulatory protein and has a key role in viral replication, pathogenicity and escape from immune responses. Analyzes of HIV sequences revealed that this protein, as well as P24, has multiple conserved and immunogenic epitopes that are recognized by cytotoxic and T helper lymphocytes (14-17). So, considering those properties and bioinformatic analysis results, fusion of P24 and Nef for candidate vaccine design was considered (9). Herein, a fusion peptide candidate vaccine based on the most conserved and immunogenic sequences in proteins from HIV P24 and Nef were used as a model to study the lasting of immune responses. Here, for the first time we have shown that immunization of mice with a mini vaccine could generate long-lived memory responses, as it was sustained 20 weeks after the final shot.

## Material and methods

**Peptide**

HIV-1 P24-Nef fusion peptide from P24 (aa_159-173_) and Nef (aa_102-117_) with a three amino acid linker comprising AAY (alanine-alanine-tyrosine) was synthesized (EPFRDYVDRFYKTLRAAYHS-QRRQDILDLWIYHT) according to the solid- phase method by GL Biochem Company (Shanghai, China) with purity >95%. Peptide was resolved in sterile phosphate buffer saline in a final concentration of 5 mg/ ml and stored at -70 ˚C until use.

**Cell Line**

P815 cell line (mouse mastocytoma, H2d) was prepared from National Cell Bank of Iran Pasteur Institute of Iran (Tehran, Iran) and cultured in RPMI 1640 (Gibco, Germany) supplemented with 10% fetal bovine serum (Gibco, Germany), 2 mM L- glutamine (Gibco, Germany), 25 mM HEPES (Sigma, USA), 0.1 mM non- essential amino acid (Gibco, Germany), 1 mM sodium pyruvate (Gibco, Germany), 50 µM 2ME (Sigma, USA), 100 µg/ml streptomycin, 100 IU/ml penicillin (Gibco, Germany) and incubated at 37 ˚C in 5% CO2 with 95% humidity.

**Mice**

Six to eight weeks old female inbred BALB/c mice were purchased from the Pasteur Institute (Karaj, Iran) and were housed under standard conditions for one week before the experiment. All experiments were in accordance with the animal care and use protocol of Tarbiat Modares University of Iran.

**Mice immunization and experimental groups**

Experimental mice groups were immunized subcutaneously (SC) with 20 µg of adjuvanted p24-Nef fusion peptide (n=18) in incomplete Freund’s adjuvant on day 0 in a total volume of 200 µl. On the days 28, 56 and 84 mice were boosted with same injection. As control group, the mice were injected with 200 µl of sterile PBS (n=15) and as adjuvant control, mice were injected with adjuvant alone (n=9) with the same protocol. For each immunoassay in each stage (weeks 4, 12 and 20 of study) 6 mice from HIV-1 P24-Nef vaccinated group, 5 mice from PBS control group and 3 mice from adjuvant control group were used.

**Lymphocyte proliferation assay**

Under sterile conditions, the mice spleens were removed and suspended in cold PBS containing 2% FBS. Red blood cells were lysed with lysis buffer and single- cell suspension was adjusted to 4×10 ^6^ cell/ml in RPMI 1640 (Gibco) supplemented with 5% FBS, 4 mM L-glutamine, 25 mM HEPES, 0.1 mM non essential amino acid, 1 mM sodium pyruvate, 50 µM 2ME, 100 µg/ml streptomycin and 100 IU/ml penicillin. 100 ml of cell suspensions were dispensed into 96- well flat- bottom culture plates (Nunc) and were stimulated with 2 μg of the peptide. Phytohemagglutinin-A (PHA) (5 μg/ml; Gibco, Germany) was used as a positive control and un-stimulated wells were used as negative controls. After incubating for 72 h at 37 ˚C in 5% CO_2_ humid incubator, cells were pulsed with 0.7 µCi of (^3^H) Thymidine (Amersham, UK) per well and incubation continued for 18 h and then cells were harvested and radioactivity was measured by beta counter (Pharmacia, Sweden). Stimulation index (SI) was calculated according to formula: cpm of the wells stimulated with antigen/cpm of the negative control wells.

**Granzyme B production assay**

Cytotoxicity assay was performed using mouse Granzyme B ELISPOT kit according to the manufacture^’^s (R&D systems, USA) instruction. Briefly, PVDF 96-well plate (Millipore, USA) was coated with Granzyme B capture antibody overnight at 4 ˚C. The plate was washed 4 times with PBS-Tween 20 (PBS-T20) and blocked with PBS containing 1% BSA (Gibco, Germany) and 5% sucrose for 2 h at room temperature. P815 cell line, as target cells, was pulsed overnight with 20 µg/ml of fusion peptide. 20.000 target cells in a volume of 100 µl per well of 96-well plates were incubated with 100 µl of splenocyte suspensions that were (over night) stimulated with PHA as effector cells at 50:1 effector: target (E:T) ratios for 4 h in complete RPMI 1640 at 37 ˚C and 5% CO_2_. The wells containing un- pulsed P815 cells with splenocytes were used as specificity control. Spleen cells, P815 and RPMI 1640 were used as negative controls and recombinant mouse Granzyme B (eBioscience, USA) was used as positive control. The plates were washed five times and incubated for 18 h at 4˚C with 100 µl of 1/60 diluted anti mouse detection antibody in PBS containing BSA 1% and then the plates were washed and 100 µl of 1/1000 diluted streptavidin-conjugated alkaline phosphatase were added to each well. After final wash with washing buffer, spots were developed with adding 100 µl of BCIP/NBT substrate to each well and incubating 60 min at room temperature in the dark. The plates were then rinsed three times with distilled water and dried at 4 ˚C. Spots were counted by stereo microscope (Nikon, Japan). All experiments were performed in triplicate and the number of specific Granzyme B producing lymphocytes was calculated by subtracting the spots from stimulated wells with the specificity controls.

**Cytokine assay**

Analysis of IL-4 and IFN-γ producing cells were performed with ELISPOT method according to the manufacture^’^s (Mabtech, Stockholm, Sweden) protocol. Briefly, a total number of 1×10^6^ spleen cells were plated on each well of 96-well, PVDF plates using complete RPMI 1640. The cells were stimulated *in vitro* with 2 µg of peptide per well, PHA was used as positive control and wells containing un-stimulated cells and RPMI 1640 were used as negative controls. The plates were incubated at 37 ˚C in 5% CO_2_. After 24 h of cells stimulation, the plates were washed five times with washing buffer and then 100 µl of 1 µg/ml mAb directed to mouse IL-4 or IFN-γ in PBS containing 0.5% FBS were added to the wells and incubated for 2 h at room temperature. The plates were washed five times with washing buffer and incubated for 1 h at room temperature with 100 µl of 1/1000 diluted streptavidin-conjugated alkaline phosphatase. After final wash with washing buffer, spots were developed with adding 100 µl of BCIP/NBT substrate to each well and incubating 45 min at room temperature in the dark. The plates were then rinsed three times with distilled water and dried at 4˚C. Spots were counted by stereo microscope (Nikon, Japan). All experiments were performed in triplicate and the number of specific IFN-γ and IL-4 producing lymphocytes was calculated with subtracting the spots from stimulated wells with the un-stimulated ones.

**ELISA of specific antibodies and isotypes**

Specific antibodies were determined by indirect ELISA method. Briefly, 100 µl of 5 µg/ml of p24-Nef fusion peptide in 50 mM carbonate-bicarbonate buffer (pH 9.6) were coated into 96-well ELISA Maxisorp plates (Nunc, Naperville, IL) by 24 h incubation at 37 ˚C. The wells were washed with PBS containing 0.05% Tween 20 (washing buffer) and blocked 1 h at 37 ˚C with 5% non fat dry milk in PBS (blocking buffer). Plates were washed with washing buffer and 100 µl of 1/100 diluted sera were added to each well and incubated at 37 ˚C for 2 h. The wells were washed three times with washing buffer and incubated for 2 h with 100 µl of 1/7000 dilution of anti mouse conjugated to horse radish peroxidase (Sigma, USA). The wells were washed five times with washing buffer and incubated 30 min with 100 µl of TMB substrate in the dark and reaction was stopped with 2N H2SO4 and color density was measured at 450 nm with ELISA plate reader. For detection of specific IgG1, IgG2a and IgM subclasses, goat anti-mouse IgG1, IgG2a and IgM were used as secondary antibodies (Sigma, USA).

**Statistical analyzes**

The data were expressed as Mean± SD. All statistical analyzes were done by one-way ANOVA followed by Tukey’s test. In all of the cases, P value less than 0.05 was considered to be statistically significant.

**Fig. 1:**
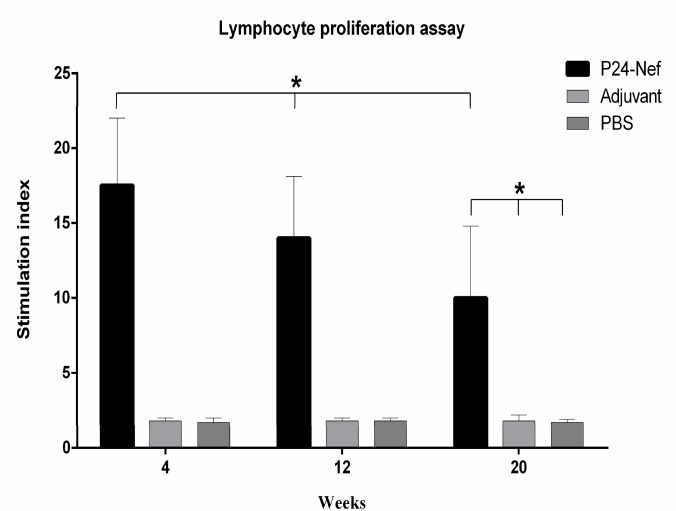
Lymphocyte proliferation response. Splenocytes of mice were harvested and stimulated *in vitro* with fusion peptide for three days and 3H thymidine radioactivity was measured at week 4 and monitored at weeks 12 and 20 after final immunization. Proliferation is presented as stimulation index of individual mice and data are presented as mean of triplicate± standard deviation (SD). Asterisks indicate the groups which were significantly different (P< 0.05

**Fig. 2:**
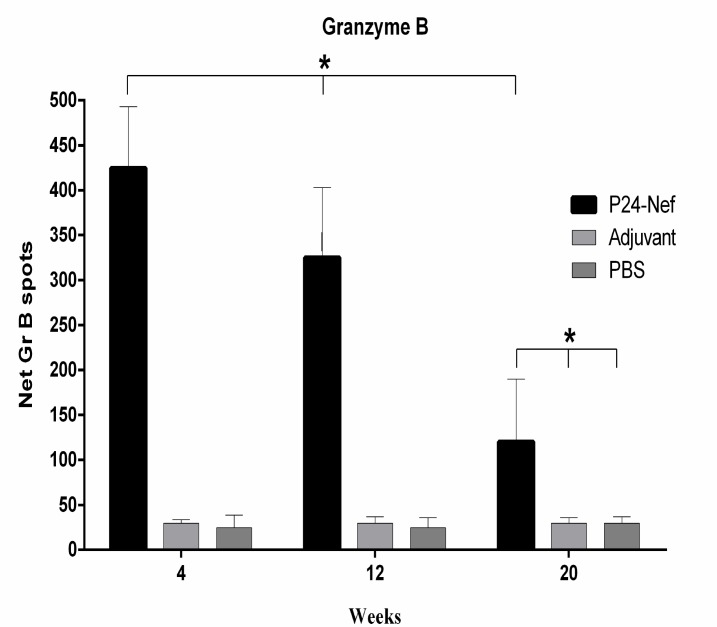
CTL response of experimental groups. Cytotoxicity of individual mice was evaluated with Granzyme B ELISPOT method. Data are presented as mean of triplicate± SD. Asterisks indicate the groups which were significantly different (P< 0.05

**Fig. 3:**
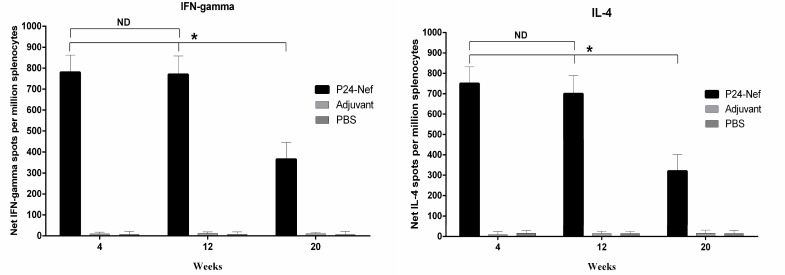
Cytokine IFN-γ and IL-4 analysis. Following immunization periods, ELISPOT was carried out to detect the frequency of IFN-γ and IL-4 cytokine producing cells. Absolute spots of individual cytokines were calculated with subtracting the corresponding negative controls. Data are presented as mean of triplicate± SD. Asterisks indicate the groups which were significantly different and ND indicates not detectable differences (P< 0.05

**Fig. 4:**
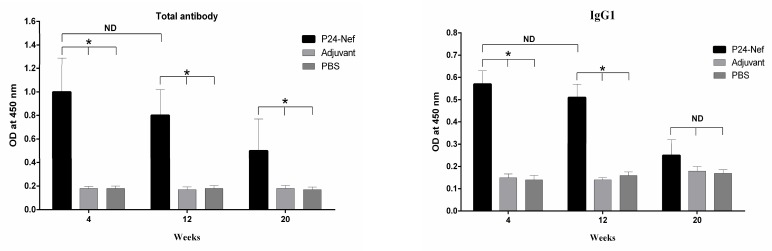
Specific humoral immune response monitoring against HIV-1 P24-Nef fusion peptide after immunization periods. (a) Sera of individual mice were collected and specific total IgG was evaluated with an optimized indirect ELISA method. Specific IgG1 (b), IgG2a (c) and IgM (d) levels were evaluated 4 weeks after final immunization. Data are presented as mean of triplicate± SD. Asterisks indicate the groups which were significantly different and ND indicates not detectable differences (P< 0.05).

## Results

**Lymphoproliferative activity**

Lymphocyte proliferative responses were monitored using *in vitro* re-stimulation of splenocytes for 72 h with 10 µg/ml of P24-Nef fusion peptide and detection of the proliferative responses with ^3^H thymidine radioactivity. As shown in figure 1, mice imm unized with P24-Nef peptide significantly induced lymphocyte prolif-eration response in comparison with control groups and this enhanced response was stable until 20 weeks after final shot (P=0.0001). No significant differences were observed between control groups (P> 0.05).

**Cytotoxic activity**

The CTL response was monitored, as a key mediator in the control of HIV infection. To detect cytotoxic activity, Granzyme B ELISPOT method was used. Analysis of GrB producing cells revealed that peptide immunization of mice increased this population compared to control groups (P=0.0001) in 50:1 of E:T ratio (Fig. 2). Monitoring of cytotoxic activity of mice immunized with P24-Nef peptide after 20 weeks revealed a decrease in CTL activity over time but significant differences were observed between immunized and control groups (P=0.0001) and no significant difference was observed between control groups (P>0.05).

**IL-4 and IFN-γ assay**

As shown in Fig. 3A, peptide immunization of mice significantly (P=0.0001) increased IFN-γ producing lymphocytes compared to control groups. This response has exhibited significance increase until the 20^th^ week of study (P<0.005). No significant difference was observed between control groups (P>0.05). We also evaluated IL-4 producing lymphocytes as Th2 cytokine marker. Evaluation of IL-4 producing cells revealed that after peptide immunization of mice, IL-4 producing lymphocytes had increased significantly (P=0.0001) compared to control groups (Fig. 3B) and this difference was stable during the study after the 20^th^ week of final immunization (P=0.0001). There was no significant difference between control groups (P> 0.05).

**Humoral immune responses and isotyping**

Humoral immune response was determined with indirect ELISA method. The results of total antibody titration showed that immunization of experimental groups with adjuvanted P24-Nef fusion peptide induced specific antibody (Fig.4a) responses. The peak of specific IgG antibody level was observed at weeks 4 (P= 0.0001) and 12 (P= 0.003) after final shot compared to control groups. At week 20 after final immunization, antibody response was detectable and showed significant difference with PBS (P= 0.009) and adjuvant (P=0.011) control groups. Furthermore, we characterized the specific isotypes of antibodies that were induced. Monitoring of IgG1 during the study revealed the highest titer in weeks 4 (P= 0.0001) and 12 (P= 0.001) of immunization (Fig. 4b). At 20^th^ week of immunization no significant difference was observed with PBS (P= 0.071) and adjuvant (P= 0.14) control groups. Monitoring of specific IgG2a antibodies revealed a peak level at weeks 4 (P= 0.0001) and 12 (P= 0.0001) of immunization compared to control groups (Fig. 4c). In week 20 of immunization, fusion peptide immunized groups exhibited significant difference (P= 0.0001) with control groups with tiny decrease in IgG2a titer. Furthermore, as shown in Fig. 4d, immunization of mice with fusion peptide induced specific IgM responses that was significantly different from PBS (P= 0.002) and adjuvant (P= 0.001) control groups.

## Discussion

Understanding the underlying mechanisms of the immune response in HIV can enable the construction of candidate vaccine from conserved and immunogenic epitopes of HIV-1, which currently seems the best way to achieve a more potent and effective vaccine with suitable memory responses (18). Utilization of multi-epitopic vaccine comprising epitopes presented by MHC I and II induced Th and CTL responses and also dual recognition of candidate vaccine by both MHC I and II contributed to the efficient stimulation of immune response and memory formation (19, 20). Here we evaluated a fusion peptide as a candidate vaccine in BALB/c mouse model and monitored immunologic parameters for memory analysis. We did not conjugate the peptide with keyhole limpet hemocyanin (KLH) or BSA because of immunomodulatory effect of conjugation (21) and did not utilize complete Freund^’^s adjuvant for immunization because of the polarization effect on specific immune responses (22, 23).

Our intention was to allow the immune response to polarize according to the nature of the fusion peptide. In the present study, our main goal was to study the long-lived memory responses following immunization with candidate peptide vaccine. So, cellular and humoral immune responses were monitored for 20 weeks after final immunization in order to evaluate specific memory T and B cell function following immunization with candidate vaccine. Analysis of T cell responses after final immunization showed that candidate vaccine exhibited enhanced proliferation activity and also, cytotoxic activity compared to control groups and this response was sustained during the study (20 weeks). These findings indicated that following immunization with candidate vaccine, long-lived memory T cells had developed and these cells exhibited *in vitro* bioactivity related to this population. Induction of long-lasting cellular immune responses following immunization guarantees the long-term protection against pathogens (24, 25) and our candidate vaccine could induce durable CTL and non CTL mediated cellular immunity. Cytokine patterns revealed that following peptide immunization, both IL-4 and IFN-γ producing lymphocytes had increased in numbers but the frequency of IFN-γ producing lymphocytes was dominant and this indicated that candidate vaccine shifted immune responses to the Th1 profile. Monitoring of cytokine responses show that after the 20^th^ week of immunization, cytokine production was sustained with Th1 dominancy and thus confirming a Th1 profile. Analysis of humoral immune responses against fusion peptide showed that the candidate vaccine induced multi-isotype humoral immune responses comprising of IgM, IgG1 and IgG2a subclasses but IgG2a was dominant.

Monitoring of humoral immune response revealed that the level of specific antibody was sustained for 20 weeks with IgG2a dominancy and this also confirmed a Th1 pattern. Induction of multi-isotype humoral immune responses may be a useful property of our candidate vaccine because of the distinct function of each isotype of antibody in humoral immune response (26). Induction of long-lived plasma cells in the bone marrow is associated with higher levels of serum antibody in the long-term period and induction of plasma cells is a Th dependent phenomenon that may be indicative of an effective induction of helper T cells which results in effective induction of antibody forming cells (26-28). Overall, our results show that immunization of HIV-1 P24-Nef fusion peptide strongly elicited B and T (CTL and non CTL mediated) lymphocytes responses and demonstrated durability in both cellular and humoral immune responses. This candidate vaccine may be employed in further animal and primate studies and in challenge studies to elucidate broader aspects of the immune response including other cytokines, chemokines and other regulatory molecules.
